# Effects of Omega-3 Polyunsaturated Fatty Acids on the Formation of Adipokines, Cytokines, and Oxylipins in Retroperitoneal Adipose Tissue of Mice

**DOI:** 10.3390/ijms25189904

**Published:** 2024-09-13

**Authors:** Tatjana Wenderoth, Martin Feldotto, Jessica Hernandez, Julia Schäffer, Stephan Leisengang, Fabian Johannes Pflieger, Janne Bredehöft, Konstantin Mayer, Jing X. Kang, Jens Bier, Friedrich Grimminger, Nadine Paßlack, Christoph Rummel

**Affiliations:** 1 Institute of Veterinary Physiology and Biochemistry, Justus Liebig University, 35392 Giessen, Germany; tatjana.wenderoth@icloud.com (T.W.); martin.feldotto@vetmed.uni-giessen.de (M.F.); jessica.hernandez-2@vetmed.uni-giessen.de (J.H.); julia.schaeffer@vetmed.uni-giessen.de (J.S.); stephan.leisengang@vetmed.uni-giessen.de (S.L.); fabian@pflieger.biz (F.J.P.); janne.e.bredehoeft@vetmed.uni-giessen.de (J.B.); 2 Center for Mind Brain and Behavior (CMMB), Universities Giessen and Marburg, 34032 Marburg, Germany; 3 Translational Neuroscience Network Giessen (TNNG), Justus Liebig University, 35392 Giessen, Germany; 4 Department of Internal Medicine, Justus Liebig University, 35392 Giessen, Germany; konstantin.mayer@innere.med.uni-giessen.de; 5 Laboratory for Lipid Medicine and Technology, Department of Medicine, Massachusetts General Hospital and Harvard Medical, Charlestown, MA 02129, USA; jxkang@mgh.harvard.edu; 6 Cardio-Pulmonary Institute, Justus Liebig University, 35392 Giessen, Germany; jens.bier@innere.med.unigiessen.de (J.B.); friedrich.grimminger@innere.med.uni-giessen.de (F.G.); 7 Universities of Giessen and Marburg Lung Center (UGMLC), Member of the German Center for Lung Research (DZL), 35392 Giessen, Germany; 8 Small Animal Clinic, Internal Medicine and Department of Veterinary Clinical Sciences, Justus Liebig University, 35392 Giessen, Germany; n.passlack@lmu.de

**Keywords:** n-3-polyunsaturated fatty acids, FAT-1-mice, white adipose tissue, oxylipins, small pro-resolving lipid mediators, adipokines, cytokines, lipopolysaccharide

## Abstract

Oxylipins and specialized pro-resolving lipid mediators (SPMs) derived from polyunsaturated fatty acids (PUFAs) are mediators that coordinate an active process of inflammation resolution. While these mediators have potential as circulating biomarkers for several disease states with inflammatory components, the source of plasma oxylipins/SPMs remains a matter of debate but may involve white adipose tissue (WAT). Here, we aimed to investigate to what extent high or low omega (n)-3 PUFA enrichment affects the production of cytokines and adipokines (RT-PCR), as well as oxylipins/SPMs (liquid chromatography–tandem mass spectrometry) in the WAT of mice during lipopolysaccharide (LPS)-induced systemic inflammation (intraperitoneal injection, 2.5 mg/kg, 24 h). For this purpose, n-3 PUFA genetically enriched mice (FAT-1), which endogenously synthesize n-3 PUFAs, were compared to wild-type mice (WT) and combined with n-3 PUFA-sufficient or deficient diets. LPS-induced systemic inflammation resulted in the decreased expression of most adipokines and interleukin-6 in WAT, whereas the n-3-sufficient diet increased them compared to the deficient diet. The n-6 PUFA arachidonic acid was decreased in WAT of FAT-1 mice, while n-3 derived PUFAs (eicosapentaenoic acid, docosahexaenoic acid) and their metabolites (oxylipins/SPMs) were increased in WAT by genetic and nutritional n-3 enrichment. Several oxylipins/SPMs were increased by LPS treatment in WAT compared to PBS-treated controls in genetically n-3 enriched FAT-1 mice. Overall, we show that WAT may significantly contribute to circulating oxylipin production. Moreover, n-3-sufficient or n-3-deficient diets alter adipokine production. The precise interplay between cytokines, adipokines, and oxylipins remains to be further investigated.

## 1. Introduction

A consequence of the modern Western diet is a shift in the ratio of omega (n)-6 polyunsaturated fatty acids (PUFAs) to n-3 PUFAs, which has evolved significantly from a ratio of 1:1 in the past to 15:1—20:1 [1]. As shown in epidemiological data, these changes are associated with an increased risk of developing chronic inflammatory conditions that can progress in severity, as has been observed for diabetes [1] or Alzheimer’s disease [2].

The well-known anti-inflammatory effects of n-3 PUFAs [3] have primarily been demonstrated in cell culture systems [4,5,6] and by evaluating serum [7,8,9] or adipose tissue after n-3 PUFA supplementation [10,11]. The anti-inflammatory pathways of n-3 PUFAs include direct action via the inhibition of arachidonic acid (AA) metabolism [12], anti-inflammatory signaling via peroxisome proliferator-activated receptors (PPARs), or the inhibition of nuclear factor-κB (NFκB) and toll-like receptor (TLR) activation [13]. These processes indirectly decrease the expression of inflammatory mediators like pro-inflammatory cytokines [14], eicosanoids, and reactive oxygen species, as well as attenuate the infiltration of immune cells through the decreased production of adhesion molecules [12,15]. For example, the inhibitory effect of eicosapentaenoic acid (EPA) on the NFκB pathway and, thus, on the production of tumor necrosis factor α (TNFα) was demonstrated in human monocytic cell culture experiments during lipopolysaccharide (LPS)-induced inflammation by the reduced degradation of the inhibitor of NF-κB (IκBα) [13,16].

Mechanisms of anti-inflammatory action of membrane-bound n-3 PUFAs like EPA and docosahexaenoic acid (DHA) are partially mediated via the production of their metabolites, so-called specialized pro-resolving mediators (SPMs) [3]. SPMs are secreted over an extended period of time during inflammation and actively contribute to its resolution and the restoration of homeostasis [17]. Oxylipins are the umbrella term for oxygenated lipid molecules derived from both n-6 and n-3 PUFAs, while SPMs are a subgroup of anti-inflammatory mediators that are important for the resolution of inflammation specifically derived from n-3 PUFAs. SPMs include lipoxins, protectins, and resolvins (Rv) [18]. Derived from EPA, remodeling via the intermediate 18-hydroxyeicosapentaenoic acid (18-HEPE) leads to the generation of the E-series Rv [17], wherein 18-oxo-RvE1 is the inactive metabolite formed locally from RvE1 by macrophages [19,20]. Instead, DHA is first converted into the intermediates 17(S)-hydroperoxy-DHA (HpDHA) and 17(S)-hydroxy DHA (HDHA) to form D-series Rvs, like RvD1 and RvD2 [17]. 17(S)-HpDHA can also be converted to protectins [21,22,23,24]. Further processing of the DHA derivative 14(S)-HpDHA [25] will produce maresin-1 [26] and 14(S)-HDHA [25]. Dose-dependent properties of these SPMs can be collectively summarized as pro-resolving and anti-inflammatory, for example, by acting via G-protein-coupled receptors to modulate neutrophil infiltration to enhance macrophage efferocytosis, which involves the removal of dead cells from the inflammatory milieu, and by the down-regulation of NFκB signaling [27,28,29]. Increased efferocytosis of apoptotic polymorphonuclear leukocytes (PMNs) [23] and control of miRNAs related to acute inflammation also belong to the properties of SPMs [30].

Various studies have already demonstrated the inflammation-resolving properties of SPMs in several diseases, including a mouse model of colitis [31], hepatitis in human and animal models [32], mouse models of airway inflammation [33], and human cardiovascular diseases [34]. For example, DHA-derived docosatrienes and 17(S)-series oxylipins reduced PMN numbers by up to 40% in peritoneal exudate in a mouse model of intraperitoneal (i.p.) injection of zymosan A-induced peritonitis and intravenous (i.v.) instillation of Rvs [21]. Oxylipin production in the brain has also been shown by studies using in vitro or in vivo n-3 PUFA enrichment approaches [35,36,37].

It is now established that adipose tissue is an endocrine organ capable of secreting a vast amount of plasma mediators, namely adipocytokines, which are involved in various processes, including chronic inflammation [38]. Adipocytokines are all the factors produced by adipocytes, which include cytokines, adipokines, chemokines, hormones, and complement factors [39]. As such, white adipose tissue (WAT) may also contribute to the production of plasma oxylipins/SPMs levels. Adipocytes represent a local store of fatty acid [40] and, together with the stromavascular fraction, are responsible for the production of adipokines [41,42]. These soluble mediators can act on various organ systems, including the brain, and also play a role in inflammation [41,42,43]. In particular, the release of adipokines involves paracrine communication between immune cells and adipocytes and local lipolytic signals from adipocytes [40]. Leptin is one pro-inflammatory adipokine, synthesized mainly in adipocytes [41]. The expression of leptin represents a link between energy balance and the organism’s defense reaction to systemic inflammation [44,45]. Leptin, thus, serves as a mediator of systemic infections, which become exacerbated as fat mass increases [45]. In addition, circulating leptin can be increased through cytokine actions and acute inflammation [46]. Adiponectin, in contrast, is rather known for its anti-inflammatory potential and can positively influence phagocytosis, suppress TNFα release [41,47], and induce the production of the anti-inflammatory cytokine IL-10 [41]. During systemic inflammation, adiponectin is able to suppress the NFκB signaling pathway and thus suppress the expression of downstream cytokines such as IL-6 and TNFα [48]. C1q/TNF-related protein-3 (CTRP3) shows its anti-inflammatory effect by inhibiting TLR4-mediated signaling pathways [49,50]. Notably, expression is increased during pre-adipocyte differentiation and is regulated by the PPARs-response element [51]. In addition, classical cytokines belong to the group of WAT-derived mediators. For example, WAT contributes to circulating IL-6 and up to 30% of plasma TNFα during systemic inflammation [52]. Overall, adipokines are released into circulation, which is further altered during systemic inflammation [44]. Adipose tissue is, thus, actively involved in the inflammatory process by producing pro- and anti-inflammatory mediators [53]. Interestingly, when incubated with n-3 PUFAs, adipocytes show increased expression of adiponectin [4]. Moreover, Bargut et al. (2017) revealed an increase in adiponectin and a decrease in TNFα and IL-6, contributing to a reversal of the detrimental effects of a high-fructose diet in mice fed a diet containing EPA or DHA for three weeks [11].

Here, we aimed to investigate how n-3 PUFAs influence the inflammatory response (i.e., the production of adipokines, cytokines, and oxylipins) in WAT upon systemic LPS-induced inflammation. For this purpose, we utilized WAT derived from genetic n-3 PUFA-enriched FAT-1 mice compared to wild-type (WT) controls 24 h after LPS injection (i.p., 2.5 mg/kg) to induce a severe systemic inflammatory response as previously reported [54,55]. FAT-1 mice endogenously synthesize n-3 PUFAs through a *C. elegans*-derived desaturase [56], allowing for a supply of n-3 PUFAs similar to dietary levels without confounding factors that may occur with oil supplementation, as previously shown [5,8,57]. Moreover, to model n-3 PUFA deficiency, FAT-1 and WT mice were kept under a standard diet with a balanced supply of fatty acids (here referred to as sufficient diet) or under an n-3 PUFA-deficient diet (here referred to as deficient diet) (Table 1). The WAT mRNA expression of the LPS receptor TLR4, pro-inflammatory signaling via NFκB activation, as indicated by IκBα expression [58], IL-6, and the adipokines leptin, adiponectin, and CTRP3 were investigated by RT-PCR to assess the LPS-induced production and alterations by n-3 PUFA enrichment or deficiency. The content of lipids and lipid metabolites, including SPMs and their precursors (oxylipins), was analyzed using liquid chromatography-tandem mass spectrometry (LC-MS/MS), as previously shown [59]. We focused on the substrates derived from AA, DHA, and EPA, namely leukotriene (LT)B4, 18-HEPE, RvE1, 18-oxo-RvE1, 14(S)-HDHA, 17(S)-HDHA, RvD1, and RvD2, as these have been shown to be of particularly high relevance for the resolution of inflammation [21,27,28,60]. AA is converted via lipoxygenases (LOXs) and intermediate steps to LTs, or via cyclooxygenases (COX) to prostaglandins, both of which are mostly pro-inflammatory [61]. By studying WAT in FAT-1 transgenic versus WT mice, we were able to gain new insights into the integrated interaction of inflammatory signaling, adipokines, and oxylipins along with their therapeutic potential and as an important source for circulating mediators including oxylipins [62,63].

## 2. Results

We focused on the questions of to what extent WAT is a source of plasma oxylipins/SPMs and how the accumulation of n-3 PUFAs affects the production of cytokines, adipokines, and oxylipins. For the demonstration of n-3 PUFA genetic enrichment, sufficient supply, and deficiency, we used a genotype of mice (FAT-1) that is capable of endogenously converting n-6 to n-3 PUFAs. A WT mouse strain functioned as a control. Additionally, we fed an n-3 PUFA-sufficient diet and compared it with an n-3 PUFA-deficient diet. Inflammatory mediators released from the WAT during LPS-induced systemic inflammation (i.p., 2.5 mg/kg, 24 h) were studied to better understand their role during the resolution of inflammation.

### 2.1. mRNA Expression of TLR4, TLR4 Signaling, and IL-6 in WAT

**Figure 1:**
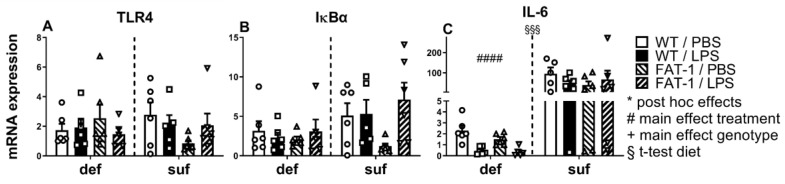
Changes in mRNA expression of inflammatory marker genes. Expression of inflammatory marker genes in white adipose tissue (WAT) 24 h p.i. with lipopolysaccharide (LPS, i.p., 2.5 mg/kg) or phosphate buffered saline (PBS). TLR4: toll-like receptor 4 (**A**); IκBα: inhibitor of nuclear factor κB (**B**); IL-6: interleukin-6 (**C**). WT: wild-type; FAT-1: transgenic n-3 PUFA-enriched mice; def: n-3 PUFA-deficient diet; suf: n-3 PUFA-sufficient diet. Analyzed by two-way ANOVA (treatment, genotype), separately for def and suf; represent mean ± SEM with symbols indicating individual mice (*n* = 5–6). #: Main effect of treatment (LPS vs. PBS) separately analyzed for def or suf. §: Effect of diet analyzed by *t*-test comparing suf with def diet regardless of their genotype or treatment. §§§: *p* < 0.001, ####: *p* < 0.0001.

We first revealed that the mRNA expression of neither the LPS-receptor TLR4 (Figure 1A) nor the indicator for NFκB activation IκBα (Figure 1B) was altered by treatment, genotype, or diet in WAT samples collected 24 h after in vivo LPS stimulation (2.5 mg/kg). In contrast to this, there was a response characterized by the higher mRNA expression of the NFκB target gene IL-6 under the n-3 PUFA-sufficient diet compared to the deficient diet (*p* < 0.001). Moreover, the main effect of LPS significantly reduced IL-6 mRNA expression, but only under the n-3 PUFA-deficient diet (*p* < 0.0001) (Figure 1C).

In summary, at the time point investigated, we only detected LPS-induced changes for IL-6 expression under the n-3 PUFA-deficient diet. We also found evidence of overall lower IL-6 expression in WAT of mice fed with an n-3 PUFA-deficient diet compared to a diet sufficient in n-3 PUFAs at the time point investigated (24 after LPS stimulation). The fact that we did not detect an LPS-induced increase in NFκB signaling suggests that inflammatory signaling may have been dampened during the course of septic-like inflammation, with potential differences in overall dynamics between groups that remain to be investigated.

### 2.2. mRNA Expression of Leptin, Adiponectin, and CTRP3 in WAT

**Figure 2:**
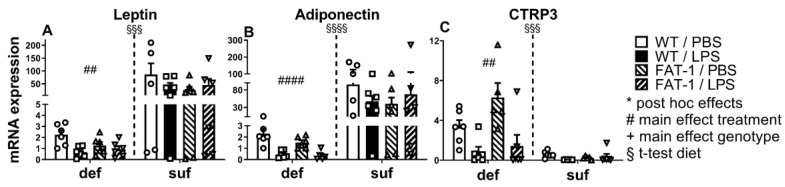
Changes in adipokine mRNA expression in WAT. Expression of white adipose tissue (WAT) adipokine levels 24 h p.i. with lipopolysaccharide (LPS, i.p., 2.5 mg/kg) or phosphate buffered saline (PBS). Leptin (**A**); adiponectin (**B**); CTRP3: C1q/TNF-Related Protein 3 (**C**). WT: wild-type; FAT-1: transgenic n-3 PUFA-enriched mice; def: n-3 PUFA-deficient diet; suf: n-3 PUFA-sufficient diet. Analyzed by two-way ANOVA (treatment, genotype), separately for def and suf; bars represent mean ± SEM with symbols indicating individual mice (*n* = 5–6). #: Main effect of treatment (LPS vs. PBS) separately analyzed for def or suf. §: Effect of diet analyzed by *t*-test comparing suf with def diet regardless of their genotype or treatment. ##: *p* < 0.001, §§§: *p* < 0.001, ####/§§§§: *p* < 0.0001.

In the following, we analyzed the effect of in vivo LPS stimulation (2.5 mg/kg, 24 h) on the mRNA expression of the adipokines leptin, adiponectin, and CTRP3 in WAT. Interestingly, there was a main effect of LPS in the mRNA expression of leptin (*p* < 0.01) (Figure 2A), adiponectin (*p* < 0.0001) (Figure 2B), and CTRP3 (*p* < 0.01) (Figure 2C) in mice fed with an n-3 PUFA-deficient diet. Moreover, increased leptin (*p* < 0.001) (Figure 2A) and adiponectin mRNA expression (*p* < 0.0001) (Figure 2B) were determined in the n-3 PUFA-sufficient diet compared to the deficient diet, while CTPR3 was elevated in the n-3 PUFA-deficient diet group (*p* < 0.001) (Figure 2C).

Overall, diet differentially altered adipokine mRNA expression. Interestingly, adiponectin was higher in the n-3 PUFA-sufficient diet group, while CTRP3 was lower, even though both are known as anti-inflammatory adipokines. LPS-induced reductions of adipokine expression in mice fed an n-3 PUFA-deficient diet reflect changes in IL-6 mRNA expression, i.e., local inflammation, suggesting differences in the dynamics between mice fed an n-3 PUFA-deficient or sufficient diet.

### 2.3. The Ratio between n-6 and n-3 PUFAs, AA, and Its Derivative LTB4 in WAT

**Figure 3:**
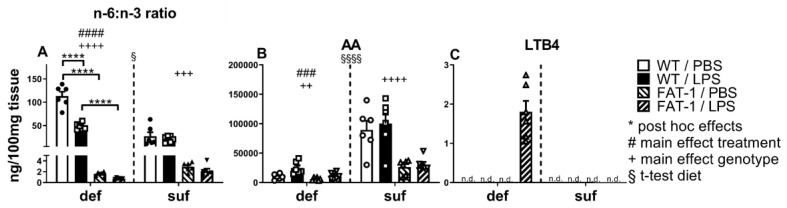
The n-6:n-3 PUFA ratio and n-6 lipid mediators in WAT. Detection and quantification of arachidonic acid (AA) and its metabolites by liquid chromatography–tandem mass spectrometry in white adipose tissue (WAT) 24 h p.i. with lipopolysaccharide (LPS, i.p., 2.5 mg/kg) or phosphate-buffered saline (PBS). n-6 (AA):n-3 (EPA+DHA) ratio (**A**); AA (**B**); leukotriene B4 (LTB4) (**C**). n-6: Omega-6; n-3: Omega-3; WT: wild-type; FAT-1: transgenic n-3 PUFA-enriched mice; def: n-3 PUFA-deficient diet; suf: n-3 PUFA-sufficient diet. Analyzed by two-way ANOVA (treatment, genotype), separately for def and suf; Šídák post hoc tests were conducted if a significant interaction was detected; interaction n-6:n-3 ratio deficient group *p* < 0.0001. Bars represent mean ± SEM with symbols indicating individual mice (*n* = 5–6). n.d.: not detected. *: Post hoc effects of treatment and genotype. +: Main effect of genotype (FAT-1 vs. WT) separately analyzed for def or suf. #: Main effect of treatment (LPS vs. PBS) separately analyzed for def or suf. §: Effect of diet analyzed by *t*-test comparing suf with def diet regardless of their genotype or treatment. §: *p* < 0.05, ++: *p* < 0.01, ###/+++: *p* < 0.001 ****/####/++++/§§§§: *p* < 0.0001.

Using LC-MS/MS, we were able to detect PUFAs and their derivatives in WAT 24 h after in vivo LPS stimulation (2.5 mg/kg). In the deficient diet, the ratio between n-6 (AA) and n-3 (EPA+DHA) PUFAs was higher in WT mice compared to FAT-1 mice (*p* < 0.0001; significant interaction with *p* < 0.0001). There was also a main effect of the genotype in the sufficient diet (*p* < 0.001) and the deficient diet (*p* < 0.0001). LPS stimulation caused the ratios to decrease in WT mice (deficient diet *p* < 0.0001) with a main effect of treatment in the deficient diet (*p* < 0.0001) (Figure 3A). As was to be expected from these results, there was a main effect of LPS in the pro-inflammatory n-6 PUFA AA, as it was increased in WAT in the LPS-stimulated groups (deficient diet *p* < 0.001). The main effect of genetic n-3 PUFA enrichment (FAT-1) resulted in significantly lower levels of AA in FAT-1 WAT compared to WT with the sufficient diet (*p* < 0.0001) and deficient diet (*p* < 0.01), confirming reduced n-6 PUFA in FAT-1 mice as previously reported [56]. Moreover, AA levels were higher in n-3 PUFA-sufficient diet groups than in mice under an n-3-deficient diet (*p* < 0.0001), which may be potentially linked to higher turnover in n-3 PUFA deficiency (Figure 3B). Interestingly, LTB4 was only detected in LPS-treated FAT-1 mice on an n-3 PUFA-deficient diet, suggesting that the conversion of AA to LTB4 may be attenuated when dietary n-3 PUFAs are sufficient (Figure 3C).

### 2.4. EPA and Its Derivatives 18-HEPE, RvE1, and 18-oxo-RvE1 in WAT

**Figure 4:**
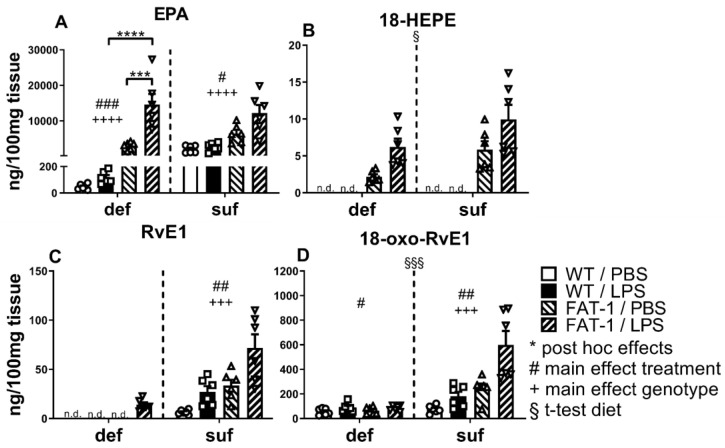
The n-3 lipid mediator EPA and its metabolites in WAT. Detection and quantification of n-3 lipid mediators and metabolites from eicosapentaenoic acid (EPA) by liquid chromatography–tandem mass spectrometry in white adipose tissue (WAT) of mice 24 h after in vivo stimulation with lipopolysaccharide (LPS, i.p., 2.5 mg/kg) or phosphate buffered saline (PBS). EPA (**A**) and its metabolites 18-hydroxyeicosapentaenoic acid (18-HEPE) (**B**), resolvin E1 (RvE1) (**C**), and 18-oxo RvE1 (**D**). WT: wild-type; FAT-1: transgenic n-3 PUFA-enriched mice; def: n-3 PUFA-deficient diet; suf: n-3 PUFA-sufficient diet. Analyzed by two-way ANOVA (treatment, genotype), separately for def and suf; Šídák post hoc tests were conducted if a significant interaction was detected; interaction EPA def group *p* < 0.001. Bars represent mean ± SEM with symbols indicating individual mice (*n* = 5–6). *: Post hoc effects of treatment and genotype. +: Main effect of genotype (FAT-1 vs. WT) separately analyzed for def or suf. #: Main effect of treatment (LPS vs. PBS) separately analyzed for def or suf. §: Effect of diet; analyzed by *t*-test comparing suf with def diet, regardless of their genotype or treatment. #/§: *p* < 0.05, ##: *p* < 0.01, ***/###/+++/§§§: *p* < 0.001, ****/++++: *p* < 0.0001.

EPA and its metabolites were analyzed by LC-MS/MS. EPA was detected in the WAT of mice 24 h after PBS or LPS stimulation. The genetic enrichment of n-3 PUFAs (FAT-1) significantly increased EPA levels in LPS-treated mice under a n-3 PUFA-deficient diet (*p* < 0.001, significant interaction with *p* < 0.001). In addition, EPA concentrations significantly increased after systemic LPS stimulation in FAT-1 mice on the deficient diet (*p* < 0.001). Genetic n-3 PUFA enrichment further increased these levels under LPS treatment (*p* < 0.0001). The main effects of treatment (*p* < 0.01) and genotype (*p* < 0.001) in the deficient group further support these findings. In the sufficient diet, there was both an effect of LPS (*p* < 0.05) and an effect of genetic n-3 enrichment (*p* < 0.0001), however, without significant interaction (Figure 4A). EPA can be enzymatically converted to 18-HEPE [17]. This derivative was detected at much lower concentrations, compared to EPA and the other measured derivatives, and only in genetically enriched FAT-1 mice. Here, a significant increase in concentration was detected in the sufficient diet compared to the deficient diet (*p* < 0.05) (Figure 4B). 18-HEPE is further metabolized to RvE1 by arachidonate lipoxygenase 5 (ALOX5) [17]. Here, RvE1 was detected in LPS-treated FAT-1 mice under an n-3 PUFA-deficient diet and in all mice under an n-3 PUFA-sufficient diet. Moreover, in the sufficient diet, RvE1 was significantly increased by LPS treatment (main effect: *p* < 0.01) and was detectable in higher concentrations in FAT-1 mice compared to WT mice (main effect: *p* < 0.001) (Figure 4C). We also detected the inactive metabolite of RvE1, namely 18-oxo-RvE1 [19,64]. Unlike RvE1, 18-oxo-RvE1 was detected in all groups. Treatment with LPS significantly increased 18-oxo-RvE1 levels in the deficient (main effect: *p* < 0.05) and sufficient diet (main effect: *p* < 0.01). Interestingly, significantly increased levels of 18-oxo-RvE1 after genetic n-3 PUFA enrichment were only detected for mice under an n-3 PUFA-sufficient diet as a main effect (*p* < 0.001) (Figure 4D).

From these results, it can be assumed that n-3 PUFAs contribute to a higher concentration of EPA and its derivatives in WAT and, thus, may facilitate the resolution of inflammation in these mice. Our data also suggest that the production or release of EPA and its metabolites are increased during LPS-induced inflammation. Overall, 18-oxo-RvE1 may represent a better marker to assess EPA metabolism to RvE1, as it was found in markedly higher concentrations than the intermediates 18-HEPE and RvE1.

### 2.5. DHA and Its Derivatives 14(S)-HDHA, 17(S)-HDHA, RvD1, and RvD2 in WAT

**Figure 5:**
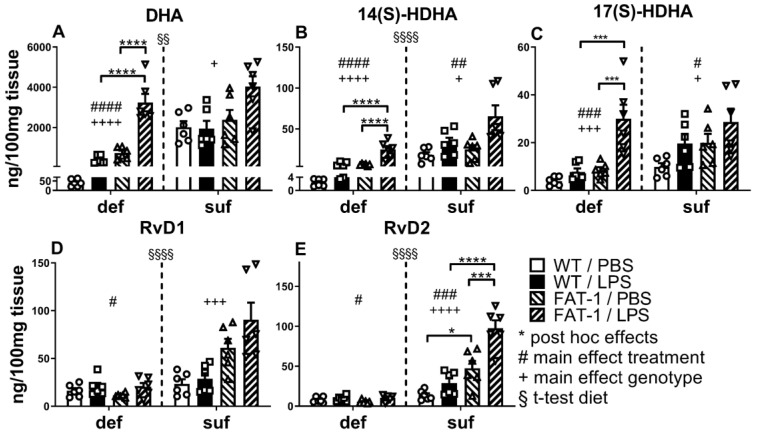
The n-3 lipid mediator DHA and its metabolites in WAT. Detection and quantification of n-3 lipid mediators and metabolites of docosahexaenoic acid (DHA) by liquid chromatography–tandem mass spectrometry. Concentrations in white adipose tissue (WAT) 24 h p.i. with lipopolysaccharide (LPS, i.p., 2.5 mg/kg.) or phosphate-buffered saline (PBS). DHA (**A**) and its metabolites 14(S)-hydroxy-DHA (14(S)-HDHA) (**B**), 17(S)-hydroxy-DHA (17(S)-HDHA) (**C**), resolvin (Rv) D1 (**D**), and RvD2 (**E**). WT: wild-type; FAT-1: transgenic n-3 PUFA-enriched mice; def: n-3 PUFA-deficient diet; suf: n-3 PUFA-sufficient diet. Analyzed by two-way ANOVA (treatment, genotype), separately for def and suf; Šídák post hoc tests were conducted if a significant interaction was detected; interaction: DHA def group: *p* < 0.001, 14(S)-HDHA def group: *p* < 0.01, 17(S)-HDHA def group: *p* < 0.05, RvD2 suf group: *p* < 0.05. Bars represent mean ± SEM with symbols indicating individual mice (*n* = 5–6). *: Post hoc effects of treatment and genotype. +: Main effect of genotype (FAT-1 vs. WT) separately analyzed for def or suf. #: Main effect of treatment (LPS vs. PBS) separately analyzed for def or suf. §: Effect of diet analyzed by *t*-test comparing suf with def diet regardless of their genotype or treatment. */#/+: *p* < 0.05, ##/§§: *p* < 0.01, ***/###/+++: *p* < 0.001, ****/####/++++/§§§§: *p* < 0.0001.

DHA was detected by LC-MS/MS, along with a large number of metabolites. The tendencies described for EPA and its derivatives were similarly observed for DHA and its derivatives. DHA itself was detected in lower concentrations than EPA. Again, a sufficient supply of n-3 PUFAs through diet proved to be an important factor for an increased occurrence of DHA (*p* < 0.01), 14(S)-HDHA, RvD1, and RvD2 (*p* < 0.0001). For DHA, the genotype had an impact in the sufficient diet, leading to a significant increase regardless of the LPS challenge (main effect *p* < 0.05). In the n-3 PUFA-deficient diet group (interaction *p* < 0.001), the FAT-1 genotype shows a strong DHA increase, while LPS significantly enhanced this effect further (*p* < 0.0001). We also observed a main effect of genotype in the deficient diet groups (*p* < 0.0001). In addition, the LPS challenge in deficient diet groups resulted in significantly higher concentrations of DHA (main effect *p* < 0.0001) and in FAT-1 mice (*p* < 0.0001) (Figure 5A), as well as higher concentrations of the other derivatives [65], such as 14(S)-HDHA (*p* < 0.0001) and 17(S)-HDHA (*p* < 0.001). These substances occurred in lower concentrations than DHA, with 17(S)-HDHA as the smallest fraction. However, both substances showed higher concentrations in genetically n-3 PUFA-enriched (FAT-1) mice after LPS treatment with the deficient diet (14(S)-HDHA: *p* < 0.0001; 17(S)-HDHA: *p* < 0.001). 14(S)-HDHA (Figure 5B) and 17(S)-HDHA (Figure 5C) were increased, as indicated by the main effects of LPS (14(S)-HDHA: def: *p* < 0.0001, suf: *p* < 0.01; 17(S)-HDHA: def: *p* < 0.001, suf: *p* < 0.05) and genotype (14(S)-HDHA: def: *p* < 0.0001; 17(S)-HDHA: def: *p* < 0.001; both suf: *p* < 0.05), however, without significant interaction in sufficient diet groups. In both sufficient diet groups, a significant interaction was observed (14(S)-HDHA (*p* < 0.01); 17(S)-HDHA (*p* < 0.05)). 17(S)-HDHA can be converted via ALOX5 to the D-series Rvs (D1-D6) [17], of which we studied D1 and D2. Both showed higher concentrations than their precursor 17(S)-HDHA. The concentration of RvD1 was increased as indicated by the main effect of the LPS challenge under the deficient diet (*p* < 0.05). Moreover, RvD1 was found in an increased concentration in the FAT-1 genotype under an n-3 PUFA-sufficient diet (*p* < 0.001) (Figure 5D). For RvD2, this main effect of genotype was also detectable in the n-3 PUFA-sufficient diet (*p* < 0.0001). Subsequent post hoc tests revealed a significant effect of genotype in the sufficient group (*p* < 0.05; significant interaction with *p* < 0.05), which was further enhanced by LPS challenge (*p* < 0.001; LPS induced increase in RvD2). Moreover, the main effect of diet for both diets (def: *p* < 0.05, suf: *p* < 0.001) supports these findings (Figure 5E).

In summary, the results were similar to those of EPA and confirmed the FAT-1 genotype in terms of increased n-3 PUFA production.

In summary, the majority of PUFAs and their derivatives were increased in expression by an n-3 PUFA-sufficient diet and LPS treatment. Moreover, the FAT-1 genotype mostly resulted in increased concentrations except for AA, where especially the WT groups showed higher concentrations.

## 3. Discussion

Overall, our study emphasizes the significant impact of n-3 PUFAs on LPS-induced inflammatory processes in WAT, which may contribute to the systemic response via circulating mediators. Mice fed with a diet containing a sufficient amount of n-3 PUFAs showed significantly increased levels of adiponectin, leptin, and IL-6 but a lower mRNA expression of CTRP3 in WAT compared to mice fed an n-3 PUFA-deficient diet (Figure 1 and Figure 2). Additionally, the n-3 PUFA-sufficient diet significantly elevated the levels of PUFAs (AA, DHA) and the respective derivatives (EPA: 18-HEPE, 18-oxo-RvE1; DHA: 14(S)-HDHA, RvD1, RvD2) in WAT (Figure 3, Figure 4 and Figure 5). As expected, mice with a genetic enrichment of n-3 PUFAs (FAT-1) showed a lower n-6:n-3 PUFA ratio, meaning enhanced levels of DHA and EPA derivatives in WAT, while AA concentrations were decreased compared to WT mice with the deficient diet. Importantly, 14(S)-HDHA, 17(S)-HDHA, and RvD2 were increased by LPS treatment in WAT compared to PBS-treated controls in genetically n-3 enriched FAT-1 mice, suggesting their potential release into circulation during LPS-induced systemic inflammation.

WAT is a primary site for energy storage [66] but is also well-known as an endocrine organ involved in adipokine secretion linked to low-grade inflammation [67]. Moreover, stimulation with LPS induces a robust inflammatory response and modulates adipokine secretion [68,69]. As such, we and others previously assessed LPS-stimulated cytokine/adipokine release using ex vivo WAT explant cultures in rats, mice, and dogs [44,70,71]. However, the effects of n-3 PUFA genetic enrichment and sufficient or deficient diet on the LPS-induced expression of adipokines and cytokines in WAT remain unclear. Todoric et al. (2006) revealed that mice with inflammation induced by a high-fat diet show reduced macrophage infiltration and diminished inflammatory gene expression in gonadal adipose tissue following 6 weeks of n-3 PUFA supplementation [72]. Moreover, supplementation with n-3 PUFAs has previously been shown to attenuate the expression of inflammatory TNFα in epididymal WAT in obese C57BL/6J mice during high-fat-diet-induced inflammation [73].

### 3.1. No Significant Effects of LPS-Induced Inflammation in FAT-1 and WT Mice Fed with an N-3 PUFA-Deficient or Sufficient Diet on NFκB Signaling

n-3 PUFAs exert anti-inflammatory capacities via the modulation of important inflammatory signaling cascades leading to the release of cytokines, such as TNFα or IL-6 [74]. For instance, mice fed with an n-3 PUFA-enriched diet exhibited reduced circulating levels and reduced epididymal WAT concentrations of IL-6 compared to mice maintained on a standard diet without n-3 PUFA enrichment [75]. While WAT was not assessed, Labrousse et al. (2018) found that maternal n-3 PUFA deficiency in C57BL6/J mice exacerbated LPS-induced inflammation as assessed by cytokines like IL-6 at gestational day 17 in fetal (E17) mouse brains [76].

Here, we expand on the existing knowledge by combining an n-3 PUFA-sufficient diet with genetic enrichment and examining retroperitoneal WAT as the production site for adipocytokines and lipid mediators following LPS treatment [68,77]. Interestingly, TLR4 expression was not affected by treatment with LPS. Instead, we found that mice maintained on an n-3 PUFA-sufficient diet actually showed higher overall levels of IL-6 expression regardless of LPS treatment. Other studies revealed that DHA can decrease TLR4-dependent NFκB activation in macrophages in a dose-dependent manner [78], which may be linked to disrupted cellular surface expression of TLR4 by DHA as previously reported [78]. In addition, it has been suggested that n-3 PUFAs may inhibit the translocation of TLR4 into lipid rafts, further inhibiting NFκB activation [79]. As such, Figueras and colleagues (2011) found the decreased gene expression of TNFα and IL-6 in the muscle and WAT of diabetic rats after EPA or linoleic acid n-3 PUFA supplementation for 28 days [80,81]. Given the established anti-inflammatory effects of n-3 PUFA supplementation [3] and that a deficient diet would lack the necessary nutrients typically involved in regulating the inflammatory response [82], we expected the production of inflammatory mediators to be more pronounced in WT mice fed a deficient diet compared to FAT-1 mice fed a sufficient diet. This has previously been reported in mice that received an n-3 PUFA-deficient diet, which enhanced cytokine expression in the brain during LPS-induced systemic inflammation [76]. Other authors suggest possible explanations for such phenomena, such as a protective mechanism of TLR4 receptors, which show downregulation due to a high dose of LPS [83]. Moreover, our measures may have missed the early peak of IkBα during inflammatory responses [84]. The timing of tissue harvesting may account for the overall higher IL-6 mRNA expression in the WAT of mice fed with a sufficient compared to a deficient diet, but this remains to be investigated in future studies. In our experiment, we assessed mRNA expression but not protein levels, which may also explain some of the observed discrepancies, as mRNA expression is not always reflected in protein levels.

Interestingly, using a model of allergic lung infection, Yin et al. (2009) revealed that mice supplemented with fish oil over 10 weeks showed increased production of the pro-inflammatory cytokines IL-5 and IL-13 [85], which are typically mitigated by the formation of SPMs [28]. These SPMs, like Rvs, maresin, and protectins, have been suggested to restore homeostasis after inflammation and limit acute inflammatory processes [86]. Clària Dalli et al. (2012) further revealed that RvD1 and RvD2 decreased pro-inflammatory TNFα and IL-6 and increased adiponectin secretion in cell cultures of adipocytes and adipose tissue explants in a model of diet-induced obesity in mice [36]. In our experiments, we found that genetic n-3 PUFA enrichment did not significantly influence the expression of inflammatory mediators. In addition, we expected an increase in inflammatory markers due to the LPS challenge, but we were unable to demonstrate any effect. Our previous data applying WAT explant cultures clearly indicated an LPS-induced secretion of IL-6 and TNFα [44,77]. However, the effects of a systemic LPS challenge on cytokine expression are dose- and time-dependent [78,82], which may reflect observed discrepancies [87]. While we have specifically chosen the dose and timing of the LPS injection, additional time points and LPS doses should be tested in future studies to further assess the dynamics of lipid mediator production in WAT. As an additional interesting perspective, the investigation of various systemic stimuli beyond LPS should be considered in future studies.

### 3.2. Diet Affects the Concentration of Adipocytokines

As a result of both in vitro and in vivo experiments performed in rodent and human subjects, n-3 PUFAs enhanced the expression and secretion of adipokines [88,89]. For instance, an in vivo feeding trial where male Wistar rats were supplemented with EPA over 5 weeks demonstrated that the expression and production levels of leptin and adiponectin in WAT from high-fat fed rats were increased by EPA enrichment compared to the control group [90]. The same research group was also able to detect an increased leptin expression and secretion in vitro within isolated rat adipocytes after incubation with EPA at doses of 10 (only elevated secretion), 100, and 200 µM over 96 h [87]. Considering the effect of LPS on the expression of adipokines, studies have also reported that during LPS-induced systemic inflammation, leptin levels are increased [91]. Moreover, it has been shown that the adipokine CTRP3 has anti-inflammatory effects on WAT in vitro by antagonizing TLR4 on adipocytes, reducing the secretion of IL-6 and TNFα [50] and attenuating NFκB signaling [92]. Together, the existing literature identifies the capacity of n-3 PUFAs to enhance the anti-inflammatory effects of adipokines, as well as their ability to regulate lipid metabolism, thereby modulating the release of adipokines [93]. Our study is interesting in this context, as it examines the effect of LPS-induced systemic inflammation in relation to gene expression in WAT. Additionally, such data may support the assumption of a close interplay between adipokines/cytokines and n-3 PUFA-derived lipid mediators. Contrary to our hypothesis that an n-3 PUFA-sufficient diet or genetic n-3 PUFA enrichment would increase the expression of adipokines in WAT during inflammation, LPS treatment showed no effects on adipokine expression levels. Moreover, diet-induced n-3 PUFA accumulation had a larger effect than genetic enrichment. Of the measured adipokines, excluding CTRP3, feeding an n-3 PUFA-sufficient diet increased the overall expression. Interestingly, an in vitro cell culture experiment demonstrated, as it was shown in our study, the inhibition of CTRP3 expression in human and mouse 3T3-L1 adipocytes after stimulation with LPS [94]. Though our results diverge from what has been reported, this disparity could be attributed to differences in sex (both vs. males only), species (mice vs. rats [90,91] or dogs [70]), LPS dosage (2.5 mg/kg vs. 100 μg/kg [90,91]), and the analyzed time points. Indeed, timing has been shown to be a significant factor capable of influencing outcomes, as observed by Sachot et al. (2004) (see above) [91] and Leuwer et al. (2009), who found that LPS-induced inflammation (100 µg/kg or 25 mg/kg) increased leptin and adiponectin expression in the adipose tissue depots of mice at 4 h but at 24 h decreased adiponectin levels, while leptin remained unchanged in comparison to control animals [69]. An interesting aspect of adipokine profiles is also the considered localization of WAT, as Leuwer et al. (2009) found that after LPS treatment, perirenal fat had reduced levels of adiponectin in comparison to epididymal and subcutaneous adipose tissue [69]. Studies by Mazaki-Tovi et al. (2011, 2012) measured adiponectin and leptin serum levels after n-3 PUFA supplementation in cats and dogs, suggesting that n-3 PUFAs may have a regulatory role in the production of adipokines [8,95,96]. In line with those observations, our study revealed only a diet effect but no genotype effect on the adipokines measured [8,97,98]. Although anti-inflammatory CTRP3 interacts with other adipokines, its effect in combination with an n-3 PUFA-sufficient diet and genetic n-3 PUFA enrichment or deficiency remains largely unknown. As it falls within the category of adipokines [99], an increased release of CTRP3 due to n-3 PUFA enrichment can be speculated, as already shown for leptin and adiponectin [87,90]. Different kinetics in the circulation could be one possible explanation for our contrary findings since we only measured expression in WAT and not circulating serum levels. Feedback loops may inhibit CTRP3 expression, while circulating serum levels remain elevated due to the half-life of CTRP3 [96,99]. Additionally, it is likely that by selecting a later time point, we missed the LPS-induced upregulation of expression.

### 3.3. n-6:n-3 Ratio is Affected by n-3 PUFA Enrichment (Genotype and Diet) and Inflammation (LPS)

Within the FAT-1 mouse line, the conversion of n-6 PUFAs into n-3 PUFAs leads to an increased concentration of EPA and DHA and a reduction of n-6 PUFA levels such as AA and therefore a decreased n-6:n-3 PUFA ratio [56,100]. In line with these findings, FAT-1 mice demonstrated a significantly decreased n-6:n-3 PUFA ratio with the deficient diet compared to WT mice. This suggests a direct conversion of AA into n-3 PUFAs like EPA or DHA in FAT-1 mice, as was shown before by Kang and his group [56,101]. The administration of LPS led to a decreased n-6:n-3 PUFA ratio in WAT in the WT [56] group fed a deficient diet, supporting the hypothesis that n-3 PUFAs and especially SPMs are being produced increasingly as a coping mechanism during inflammation [97,102]. Other studies have already shown the anti-inflammatory effect of a low n-6:n-3 PUFA ratio on acute inflammation [56]. Moreover, Hintze and colleagues demonstrated a more prominent effect of PUFA ratio than PUFA concentration during the acute inflammatory response in mice [98].

Other interesting aspects to consider are the competition between n-3 and n-6 PUFAs for incorporation into phospholipids [103] and the reduction of n-6 PUFA release from membranes due to n-3 PUFAs [104]. Here, by feeding a diet sufficient in n-3 PUFAs, AA may be increased as a result of a reduced release of n-6 PUFAs due to the incorporation of n-3 PUFAs. In addition, n-3 and n-6 PUFAs compete not only for incorporation into the membrane but also for the converting enzymes. The shift in the ratio of n-6 to n-3 PUFAs leads to an increased conversion of n-3 PUFAs compared to n-6 PUFAs. This is further supported by the low amounts of LTB4, as AA was not further converted to its derivative. One way to support this theory could be through further plasma analysis since Balvers et al. (2012) identified a discrepancy between circulating serum levels of inflammatory mediators, like eicosanoids, and their inflammatory effects in tissues like WAT [105]. Upon examination of the PUFA composition (Table 1), it is interesting to note that AA concentrations do not differ between diets, and concentrations of linoleic acid (C 18:2), AAs’ precursor, are similar. Overall, the effect of LPS on AA could only be shown in the n-6:n-3 PUFA ratio. Other studies report increased AA levels after administration of LPS either i.p. or intratracheally in combination with a fish oil-enriched diet or in FAT-1 mice [105]. In terms of nutrition, it would be interesting to investigate other diets, such a high-fat or Western diet, in our context.

### 3.4. Effects of Diet or Genetic N-3 PUFA Enrichment on EPA, DHA, and Their Derivatives Are Stronger than LPS-Induced Effects

If membrane compositions can be altered in favor of DHA and EPA through dietary PUFA supplementation, the subsequent modulation of eicosanoid expression could cause a shift towards an anti-inflammatory profile. In our study, a sufficient supply of n-3 PUFAs was reflected in the levels of DHA as well as EPA and DHA derivatives in WAT, both of which were enhanced by genetic or diet-induced n-3 PUFA enrichment. In line with our findings, Fisk et al. (2022) have reported that individuals consuming a diet rich in n-3 PUFAs developed a different fatty acid composition in subcutaneous WAT. It had shifted in favor of the anti-inflammatory SPMs [106]. A critique of the methodology is that the PUFA α-linolenic acid (C 18:3), as a precursor for EPA and DHA, is more concentrated in the deficient diet than in the sufficient one (Table 1). Moreover, by comparing FAT-1 and WT mice supplemented with sunflower oil or maintained on standard diets, Ostermann and Waindok et al. (2017) found that both genetic and diet-induced n-3 PUFA enrichment led to an increase in EPA and DHA oxylipins in circulation and tissues [107]. In comparison to the present study, parallels emerge between the tissues analyzed by Ostermann and Waindok (plasma, brain, and colon) and our WAT. Through a sufficient dietary supply of n-3 PUFAs and through genetic n-3 PUFA enrichment as well, n-3 PUFAs lead to higher oxylipin concentrations.

Regarding EPA and its derivatives, the beneficial effects, including an anti-inflammatory lipid profile [108] and oxylipin production, are mainly observed in the FAT-1 mice under an n-3-sufficient diet. Thus, it could be argued that both n-3 PUFA-enriched groups had more inflammatory resolution following LPS stimulation. The relatively low amounts of 18-HEPE could be due to its rapid conversion to RvE1 [109], which was mainly found in mice fed a sufficient diet. Although we did not detect RvE1, we were able to detect 18-oxo-RvE1 in mice fed a deficient diet. This indicates a rapid conversion of the low amount of RvE1 to its inactive product by 15-prostaglandin dehydrogenase in these mice. Interestingly, the concentration of EPA and its derivatives increased with the sufficient diet in most cases after LPS-induced inflammation as a main effect. In this regard, studies showed that inflammatory exudates caused by LPS accumulate increased levels of SPMs in an attempt to restore homeostasis [97,102]. Indeed, an anti-inflammatory potential for oxylipins, such as Rvs, protectins, and maresins [110] was shown for the brain using mice fed diets enriched with long-chain PUFAs for two months compared to mice held on a long-chain PUFA-deficient diet after peripheral LPS-stimulation [36]. In this study, the authors revealed reduced LPS-induced pro-inflammatory cytokine production accompanied by increased n-3 PUFA-derived oxylipin levels [36]. Recently, we were able to show that oxylipins are also produced in circumventricular organs, brain structures with a leaky blood–brain barrier, prone to detecting circulating mediators such as cytokines and oxylipins during LPS-induced inflammation [35]. Interestingly, the detection of plasma oxylipins has emerged as an interesting biomarker for health and disease, including chronic inflammatory processes like Alzheimer’s disease [111,112].

Similar results to those observed for EPA were found for DHA and its derivatives since we detected higher levels with the sufficient diet, and these levels were further exacerbated in FAT-1 mice after treatment with LPS, as was demonstrated beforehand [97,102]. In general, the concentrations of EPA and its derivatives were slightly higher compared to DHA and its derivatives, suggesting a higher content of EPA compared to DHA in WAT, as was shown previously [113]. Serhan et al. (2009) discovered that 17(S)-HDHA accumulated in vitro in inflammatory exudates and could serve as a marker of 17(S)-D series Rvs and protectin biosynthesis [114]. The demonstrated increased concentration of the DHA and its derivative 14(S)-HDHA probably contributed to the enhanced adiponectin levels since these DHA-derived products induce the activation of peroxisome proliferator-activated receptor γ [115], which enhances adiponectin release from fat cells [116]. In addition, Hellmann et al. (2011) demonstrated in WAT using leptin-resistant mice, wherein RvD1 was found to increase adiponectin production while simultaneously reducing IL-6 levels [117]. The low concentrations of RvD1 and RvD2 in mice on the deficient diet could be due to low precursor conversion or a high inactivation rate of the D-series Rvs. Further investigations and measurements of different D-series SPMs like protectins and maresins and their inactive products as well can help to examine the connection between the production of oxylipins and LPS-induced inflammation. While the full physiological significance of SPMs/oxylipins being produced in WAT remains to be investigated in future studies, previous studies have already gained insights into the role of such mediators during infection and inflammation [30,64,110], highlighting their relevance as well for WAT and its secretion into circulation.

To understand the temporal course of oxylipins in different tissues, a study demonstrated how, during LPS-induced inflammation, the oxylipin concentrations of 15 different eicosanoids peaked before decreasing over time [117]. Interestingly, the oxylipin concentrations decreased more rapidly in plasma than what was observed in WAT [105]. This suggests that there are time-dependent effects that differ between circulating and tissue oxylipin levels. Thus, for a better understanding of inflammatory effects, an analysis of plasma and WAT would be informative.

### 3.5. Simultaneous Increase in Adipokines Alongside Oxylipins/SPMs Suggests an Interaction between Their Regulations

The relationship between the regulation of adipocytokines and the individual PUFAs, including their derivatives, has been explored in previous studies, e.g., in the serum and brain tissue of FAT-1 mice [103]. By demonstrating a simultaneous increase in adipokines alongside oxylipins/SPMs, it was shown that the formation of the latter is favored by adipokines. While our study did not directly confirm the connection between adipokines and oxylipins/SPMs, Zahradka et al. (2017) found that oxylipins are capable of modifying 3T3-L1 mice preadipocyte properties including their lipid metabolism and adipokine production [118]. In addition, n-3 PUFA supplementation in women has been shown to increase plasma levels of both oxylipin and adiponectin [119]. Moreover, others have shown that RvD1 and RvD2 decreased TNFα and IL-6 and increased adiponectin in cultured adipocytes and adipose tissue explants using a mouse model of diet-induced obesity [120].

In line with our findings, another study highlighted the numerous targets of n-3 PUFAs. EPA and DHA supplementation in patients with chronic inflammation not only reduced inflammatory processes in plasma (IL-6 and TNFα) but significantly altered the SPM lipidome. Moreover, derivatives of AA were replaced by the DHA and EPA derivatives and therefore decreased the n-6:n-3 PUFA ratio [121]. Indeed, 18-HEPE was increased by EPA, whereas 17- and 14(S)-HDHA were upregulated by DHA [121]. Also, in our case, the n-3 PUFA-sufficient diet demonstrates reduced inflammation (IL-6). An increase in n-3 PUFA metabolites (e.g., RvD2; 14(S)-HDHA) displaces AA and is possibly indicative of potential underlying mechanism that may explain changes in the content of mediators in WAT revealed in our present study.

This allows us to consider the effects of oxylipins that we investigated in relation to the action of adipokines. Pflieger and Wolf et al. (2022) constructed an in vitro experimental setup using rat sensory circumventricular organs in cultures enriched with n-3 and n-6 PUFAs [35]. It was demonstrated that LPS can increase (e.g., TNFα bioactivity and signaling; COX2) or decrease (epoxide hydrolase) enzymes and signaling pathways [35]. Additionally, LPS showed an increase in many n-3 PUFA oxylipins and COX2 metabolites, while there was both upregulation and downregulation observed in n-3 PUFA oxylipins [35]. Moreover, elevated levels of metabolites derived from DHA (e.g., 14(S)-HDHA; RvD2) were observed in our experiment, possibly associated with the altered signaling pathways induced by LPS. The presence of oxylipins/SPMs in the WAT and their potential as biomarkers when released into the bloodstream may be of high relevance and should be further assessed in future studies.

## 4. Materials and Methods

### 4.1. Animals

The sample material originated from a previous study, in which retroperitoneal adipose tissue samples from eight animal groups were obtained from male mice. Parental animals were obtained from Charles River Laboratories, Sulzfeld, Germany, and further breeding took place at the Justus Liebig University Giessen [97]. Besides transgenic FAT-1 mice (male B6.129P2-Tg(CAG-fat-1)Jxk backcrossed to C57BL/6N), WT mice (C57BL/6N) were used as control animals. The latter were the genetic background for breeding the transgenic FAT-1 mice. Animals were 6–8 weeks old and weighed circa 25 g. Transgenic FAT-1 mice express the *fat-1* gene of *Caenorhabditis elegans*, which encodes an n-3 PUFA desaturase that converts n-6 to n-3 PUFAs by inserting another double bond [56]. Thus, unlike other mammals, these mice can endogenously synthesize larger amounts of n-3 PUFAs [56]. The FAT-1 mice were genotyped by using ear punch samples (necessary for labeling) by gel electrophoresis. Previous studies have shown that FAT-1 mice show similar n-3 PUFA levels after feeding an n-3 enriched diet [122]. Moreover, n-3 PUFA-deficient and sufficient diets have previously been used in FAT-1 mice to gain new insights into the functional role of n-3-derived lipid mediators [123,124,125]. Here, two feeding groups were included, which consisted of a group fed an n-3-deficient diet (S8909, Ssniff Spezialdiäten GmbH, Soest, Germany) and a group fed a standard diet with a balanced supply of n-3 PUFAs (V1126, Ssniff Spezialdiäten GmbH, Soest, Germany) (Table 1 and Table 2). In both feeding groups, food and water were available ad libitum and were energetically balanced (Table 2). Already the parent animals were on their respective diets starting when a “plug” proved the presence of pregnancy. During lactation, the n-3-sufficient diet was increased to 12% crude fat for reasons of palatability and was reduced back to 7% after weaning (days 21–28). These diets were maintained until the end of experiments. During the experiment, animals were kept under controlled environmental conditions (50% ± 5% humidity, for mice thermoneutral 30 ± 0.1 °C, 12 h light/dark cycle) in type III cages (Ehret GmbH and Co. KG Labor, und Pharmatechnik, Emmendingen, Germany) in a climate chamber (supplier: Weiss Umwelttechnik GmbH, Type 10′US/+5 to +40 DU, Reiskirchen, Germany). All processes involving live animals were approved by the Giessen Regional Council (RP Giessen Az: GI 18/2 Nr. 28/2013).

### 4.2. Treatment and Sample Preparation

The mice were injected i.p. with the inflammatory stimulus LPS (*E. coli*, serotype O111:B4, Lot: 030M4114, Sigma-Aldrich Chemie GmbH, Steinheim, Germany) at a dose of 2.5 mg/kg body mass or PBS (Dulbecco’s phosphate buffered saline, PAA GmbH, Pasching, Austria); both at a volume of 5 mL/kg. The LPS treatment took place in the morning between 9:00 and 11:00 a.m. Sampling was performed 24 h after injection by deep terminal anesthesia with sodium pentobarbital (Merial GmbH, Hallbergmoos, Germany) at a dose of 60–100 mg/kg. After opening the abdomen and thorax, blood was first collected from the right ventricle, and animals were perfused with 4–6 mL of ice-cold isotonic saline (isotonic sodium chloride (NaCl)-solution 0.9% sodium chloride; B.Braun Melsungen AG, Melsungen, Germany). Retroperitoneal WAT was frozen on powdered dry ice and stored at –80 °C before further analyses. WAT samples were cut into 20 μm thick sections with a cryostat (CryoStar NX50, Thermo Scientific, Dreieich, Germany). A total of 40 mg per sample was transferred into Eppendorf tubes for RNA extraction; on average 42 mg per sample were preserved for mass spectrometry in 2ml micro-screw tubes (Sarstedt AG and Co. KG, Nümbrecht, Germany).

We chose LPS as our inflammatory model based on the studies by Seemann et al. (2017), who compared several models [126]. They concluded that LPS is the most suitable systemic infection model when interested in studying the effects of new therapies for acute inflammation. It is technically easy to apply and provides a reproducible inflammatory response [126]. Suitable doses and timing were selected according to previous studies to ensure robust inflammatory activation of WAT [44,78,127,128]. Physiological LPS plasma levels in rodents are approximately 600–1000 pg/mL [129]. These levels can rise to 10–100 ng/mL in mice through experimentally induced endotoxemia (e.g., 1.9 mg/kg [130]; 3 mg/kg [131]; 5 mg/kg [126]). In studies of bacterial infections in tissue, even higher local levels can occur [129].

### 4.3. Real-Time PCR

RNA was extracted from each adipose tissue sample using TRIzol (Invitrogen, Carlsbad, CA, USA) according to the manufacturer’s protocol. Reverse transcription was then performed with about 5 ng total RNA to obtain complementary (c)DNA using M-MLV reverse transcriptase (50 U), dNTP mix (10 mM), and random hexamers (50 μM; Applied Biosystems, Foster City, CA, USA). After photometric determination of relative cDNA quantity and quality, relative quantification was performed in duplicate in a StepOnePlus Real-Time PCR System (Applied Biosystems, Forster City, CA, USA). Applied Biosystems TaqMan Gene Expression Assays and TaqMan Gene Expression Master Mix were used.

Initially, CANX, β-actin, GAPDH, and UBC were tested as reference genes for normalization of the samples. Through NormFinder software (version 0.953, Department of Molecular Medicine, Aarhus University, Aarhus, Denmark), CANX and UBC turned out to be the best combination of two housekeeping genes according to the stability value [132]. To combine the two best housekeeping genes, the geometric mean of the best reference gene concentrations can be used to calculate the relative expression of genes [133]. For relative quantification of the expression of each gene, the 2^−(ΔΔCt)^ method was calculated so that the results represent the multiple differences compared to a control sample, which had the lowest expression. The following assay IDs (Table 3) were applied for analyses of CTRP3, IL-6, TLR4, IKBα, leptin, adiponectin, CANX, and UBC.

### 4.4. Mass Spectrometric Analysis by LC-MS/MS for Detection of the Extracted Eicosanoids and Their Derivatives

To analyze lipid mediator content in WAT, tissue was subjected to an extraction protocol, as previously described [134]. Afterwards, samples were analyzed by liquid chromatography–tandem mass spectrometry (LC-MS/MS), according to the method described by Kiss, Röder et al. (2008) [59] using the Daltonik amaZon SL (Bruker Corporation, Billerica, MA, USA) and the 1100 series capillary LC unit (Agilent Technologies Deutschland, Waldbronn, Germany). Settings are summarized in Table 4. The resulting characteristic mass spectrometry extracted ion chromatograms (EIC traces) were used for quantification (Table 5).

### 4.5. Data Analysis

For the statistical analyses, the above genes were divided into diet groups, and effects of treatment and genotype were studied separately for each group. Moreover, treatment and genotype were combined to evaluate effects of diet. Analysis of the data was performed by two-way ANOVA (Prism 9 software; GraphPad, San Diego, CA, USA). Šídák post hoc tests were conducted if a significant interaction was detected. Effects of diet were analyzed using a *t*-test comparing suf with def diet, regardless of their genotype or treatment. Outlier calculation was performed according to Grubbs’ test using GraphPad Prism. *p* < 0.05 was applied for statistical significance, and means ± SEM are displayed in the figures.

## 5. Conclusions

In conclusion, our study demonstrates that LPS-induced inflammation led to the upregulation of resolution-promoting SPMs in WAT after genetic n-3 PUFA enrichment or on an n-3 PUFA-sufficient diet. Interestingly, some derivatives seem to be better candidates for detection and may be suitable biomarkers for inflammatory processes. However, establishing a universal method for the measurement of these biomarkers is essential. It can be postulated that WAT is an important source of oxylipins/SPMs, as it can be assumed that the PUFAs enriched by feeding and the genotype act as a source for the conversion to SPMs and are released into the bloodstream.

## Figures and Tables

**Table 1:** Polyunsaturated fatty acid composition of the diet.

Fatty Acid	n-3 PUFA-Deficient Diet	n-3 PUFA-Sufficient Diet
C 14:0	0.80%	0.01%
C 16:0	0.66%	0.56%
C 18:0	0.56%	0.14%
C 20:0	0.02%	0.02%
C 18:1	0.38%	0.96%
C 18:2	2.26%	2.42%
C 18:3	0.02%	0.31%

Data according to the manufacturer in % of the total diet.

**Table 2:** Polyunsaturated fatty acid composition of the diets.

Ingredients	n-3 PUFA-Sufficient Diet	n-3 PUFA-Deficient Diet
crude protein	22.1%	18.5%
crude fat	4.5%	7.1%
crude fiber	3.9%	5.0%
crude ash	6.7%	3.5%
starch	35.8%	28.9%
sugar	5.2%	14.0%
energy	15.9 ME [MJ/kg]	16.2 ME [MJ/kg]

Data according to the manufacturer in % of the total diet.

**Table 3:** Primer list.

Gene	ID	Producer
IL-6	Mm00446190_m1	Thermo Fisher Scientific Inc., Waltham, MA, USA
NFκBiα	Mm00477798_m1	Thermo Fisher Scientific Inc., Waltham, MA, USA
TLR4	Mm00445273_m1	Thermo Fisher Scientific Inc., Waltham, MA, USA
Leptin	Mm00434759_m1	Thermo Fisher Scientific Inc., Waltham, MA, USA
Adiponectin	Mm00456425_m1	Thermo Fisher Scientific Inc., Waltham, MA, USA
CTRP3	Mm00473047_m1	Thermo Fisher Scientific Inc., Waltham, MA, USA
CANX	Double-dye probe geNorm 12 gene kit	PrimerDesign Ltd., Southhampton, UK
UBC	Double-dye probe geNorm 12 gene kit	PrimerDesign Ltd., Southhampton, UK
GAPDH	Double-dye probe geNorm 12 gene kit	PrimerDesign Ltd., Southhampton, UK
β-Actin	Double-dye probe geNorm 12 gene kit	PrimerDesign Ltd., Southhampton, UK

**Table 4:** Mass spectrometer settings for the Bruker Daltonik amaZon SL.

	Settings
General	MS stage: MS/MS (MS²), MRM “on” Polarity: negative Trap: ICC “on”, Target “35.000”, Max. Accu Time “50 ms”, Scan “70 to 700 *m*/*z*”, Averages “3” Rolling Averaging: No. “3”
Mode	Scan Mode: Ultra Scan
Source	Capillary: 3600 V, End Plate Offset: 500 V, Nebulizer: 4.0 psi, Dry Gas: 2.0 L/min, Dry Temp: 80 °C
MRM	MS/MS: Isolation “on”, width “1.5”, Reaction “on”, Cut-Off Selection “default”, Smart Frag “Enhanced” (for all precursors) Segment Limit 1 (0–9.5 min): Precursor 349, 347, 375, 359 Segment Limit 2 (9.5–45 min): Precursor 335, 339, 317, 343, 359, 375 Segment Limit 3 (45–110 min): Precursor 301, 306, 327, 303, 314

**Table 5:** Compounds, retention times, and EIC MS² trace definitions.

Compound	Retention Time [min]	EIC MS²
RvE1	6.7	291; 269; 205; 195; 161—MS^2^ (349)
RvD2	7.6	277; 259; 241; 233; 215; 141—MS^2^ (375)
RvD2	8.1	277; 259; 241; 233; 215; 141—MS^2^ (375)
LTB₄-d₄ (Internal Standard—1)	11.2	197—MS^2^ (339)
LTB₄	11.2	255; 195; 181; 129—MS^2^ (335)
18-HEPE	15.9	259; 215—MS^2^ (317)
17(S)-HDHA	21.9	245; 201—MS^2^ (343)
14(S)-HDHA	23.3	205; 161—MS^2^ (343)
EPA-d₅ (Internal Standard—2)	61	262; 208—MS^2^ (306)
EPA	62.1	257; 203—MS^2^ (301)
DHA	88.6	283; 229—MS^2^ (327)
AA-d₁₁ (Internal Standard—3)	96.3	270; 216—MS^2^ (314)
AA	99.2	259; 205—MS^2^ (303)

## Data Availability

The data presented in this study are contained within the article or are available upon reasonable request from the corresponding author.
